# Lithographic Performance of Aryl Epoxy Thermoset Resins as Negative Tone Photoresist for Microlithography

**DOI:** 10.3390/polym12102359

**Published:** 2020-10-14

**Authors:** Vitor Vlnieska, Margarita Zakharova, Andrey Mikhaylov, Danays Kunka

**Affiliations:** 1 Karlsruhe Institute of Technology (KIT), Institute of Microstructure Technology, Hermann-von-Helmholtz-Platz 1, 76344 Eggenstein-Leopoldshafen, Germany; margarita.zakharova@partner.kit.edu (M.Z.); andrey.mikhaylov@kit.edu (A.M.); danays.kunka@kit.edu (D.K.); 2 Chemistry Department, Federal University of Paraná, Rua Coronel Francisco Heráclito dos Santos, 100, Jardim das Américas, Curitiba, PR 81531-980, Brazil

**Keywords:** epoxy resins, photo-resins (photoresists), UV lithography, deep ultraviolet Lithography

## Abstract

Photoresists (or photo-resins) are the main and most important raw material used for lithography techniques such as deep X-ray (DXRL), ultraviolet (UVL), deep-UV (DUVL), and extreme UV (EUVL). In previous work, we showed how complicated could be the synthesis of the resins used to produce photoresist. In this study, we follow up on the strategy of tuning deep and macro levels of properties to formulate photo-resins. They were developed from a primary basis, using epoxy resins, a solvent, and a photoinitiator in several concentrations. The formulations were evaluated initially by the UVL technique, using a squared pattern of 2.3 mm^2^. The most suitable compositions were then studied in a pattern structure varying from 50 down to 1 µm width, applying UVL and DUVL. The patterned structures were compared with the chemical composition of the photo-resins. Considering the deep level of properties, polydispersion, and epoxidation degree were evaluated. Regarding the macro level of properties, the concentration of photoinitiator was studied. Promising results have been achieved with the control of the deep and macro levels methodology. By means of UV lithography, it was possible to note, for a large feature size above 2.0 mm^2^, the formulations presented good quality structures with a broad range of epoxidation degrees and photoinitiator concentrations, respectively from 3 to 100% (mol·mol_polymer_^−1^) and from 10 to 40% (mol·mol_polymer_^−1^). For structures smaller than 50 µm width, the composition of the photo-resins may be restricted to a narrow range of values regarding the formulation. The results indicate that the polydispersion of the oligomers might be a significant property to control. There is a tendency to better outcome with a low polydispersity (resins P1 and P2). Regarding UV and deep-UV irradiation, the best results were achieved with UV. Nevertheless, for DUV, the sensitivity seems to be more intense, leading to well-defined structures with over-exposure effects.

## 1. Introduction

In the history of the photoresists, a large number of formulations and approaches were proposed; however, just a few of them are able to fulfill the requirements for the production of high aspect ratio (HAR) structures [1,2]. Epoxy-based photoresists are applied in most of the processes that involve high precision in the patterning, high exposure intensity, and HAR structures. It presents better characteristics when compared to other photoresists [3], and since the early 1970s, negative tones photoresists (photo-resins) have been the best suitable photo-resins to achieve HAR structures by lithography techniques such X-ray lithography (DXRL) and deep-UV lithography (DUVL) [4,5].

This type of material has to present a set of properties with a narrow range of variation such as sufficient photosensitivity, slow degree of shrinkage, high mechanical stability, adequate hardness after post-processing (e.g., electroplating), strong adhesion to the substrates and high chemical stability after finishing the lithographic process [6,7]. It exhibits high optical transparency for the wavelengths above 360 nm, high sensitivity to UV wavelength (350–400 nm), it is inert to several solvents, bases, acids, and it presents thermal stability after cross-linking reaction [6,8,9].

**Figure 1:**
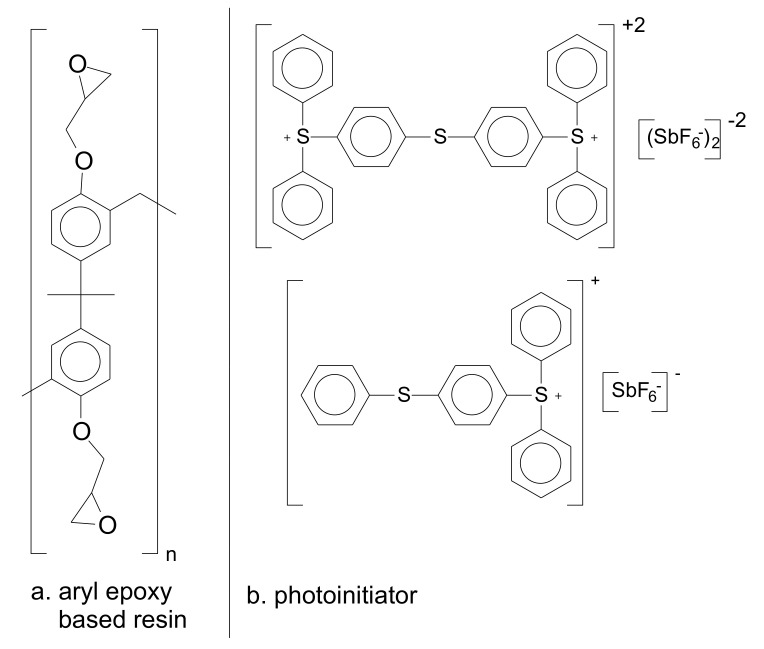
Main chemical compounds of one kind of negative-tone photoresist. (**a**) Aryl-epoxy polymer; (**b**) Triarylsulfonium hexafluoroantimonate salts.

For some of the high-end applications, e.g., optical components for interferometric techniques, the constraints, and features regarding design are very challenging. In these cases, epoxy-based photo-resins are the best alternative to be used as raw material in lithographic techniques. This photo-resin is a mixture of several compounds, however, in a simplified overview, it consists mainly of a polymer and a photoinitiator, both solubilized in a solvent. The molecular structure of the main and most used chemical compounds for the formulation of an epoxy-based photo-resin is shown in Figure 1 [10].

**Figure 2:**
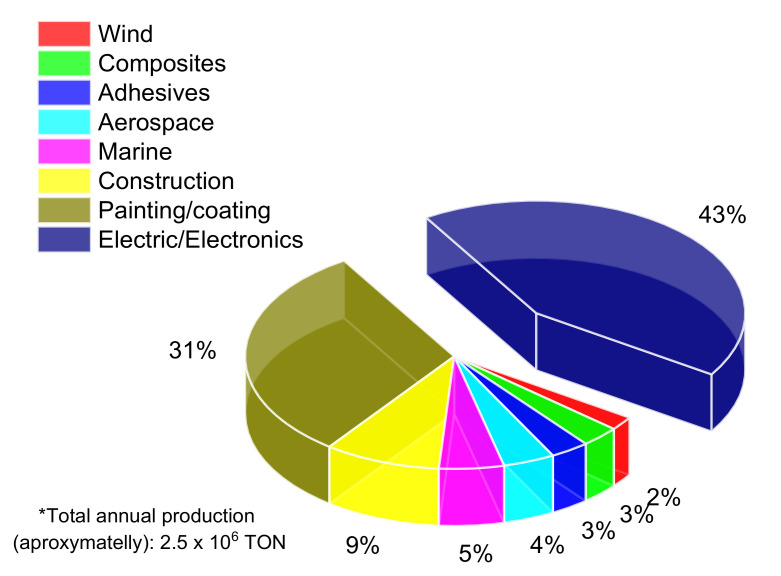
Distribution of the epoxy resins production by industrial field [11,12].

To fulfill all these requirements at the same time and in a unique material, it is necessary to consider not only the lithographic technique as a process chain but also the synthesis, characterization, and formulation of the photo-resin. Since the UVL, DUVL, and DXRL lithographic techniques are the most used to pattern structures in the range from micrometers to hundreds of nanometers (regarding periodicity) and heights up to hundreds of micrometers, specific chemical composition, and mechanical behavior from the photoresists are required. However, there is still a lack of correlation between photo-resin production and lithographic techniques such as DXRL and DUVL [5,10]. Specifically, for photoresists made from aryl-epoxy resins, one can see in Figure 2 the distribution of the production for the most important epoxy resins, regarding the application fields.

Epoxy resins are an essential class of industrial products; worldwide electric/electronic and painting/coating fields are the major industry sections utilizing this material. Although there is a limitation regarding the information on the market share for DXRL and DUVL, a rough estimation of the semiconductor manufacturing market can be suggested, the annual demand of photoresists for the North American market is approximately 1.9 × 10^3^ tons. It represents less than 0.001% of the whole epoxy resins production. Interestingly, in this approximation were considered all kinds of photoresists applied in the semiconductor industry, including the most used acrylate-based polymers [11]. It seems that photoresists for DXRL and DUVL have insignificant parcels in the market share, and perhaps this is the reason not to develop customized photoresists for such applications.

As mentioned before, one of the types of negative tone photo-resins is composed of at least three components: an epoxy-based resin, a photoinitiator, and a solvent. Other chemicals can be added to enhance the properties of the photo-resin, like adhesion promoters, inhibitors of crosslinking, sensitivity enhancers, fillers, etc. [12,13]. As described by a previous publication [10], the characteristics of the photo-resins can be tuned at least into two levels, where the definitions are: deep level (synthesis and characterization of the resins, leading to specific chemical/mechanical properties) and macro level (tuning the properties of the photo-resins by mixing other chemical compounds to obtain the final formulation).

**Figure 3:**
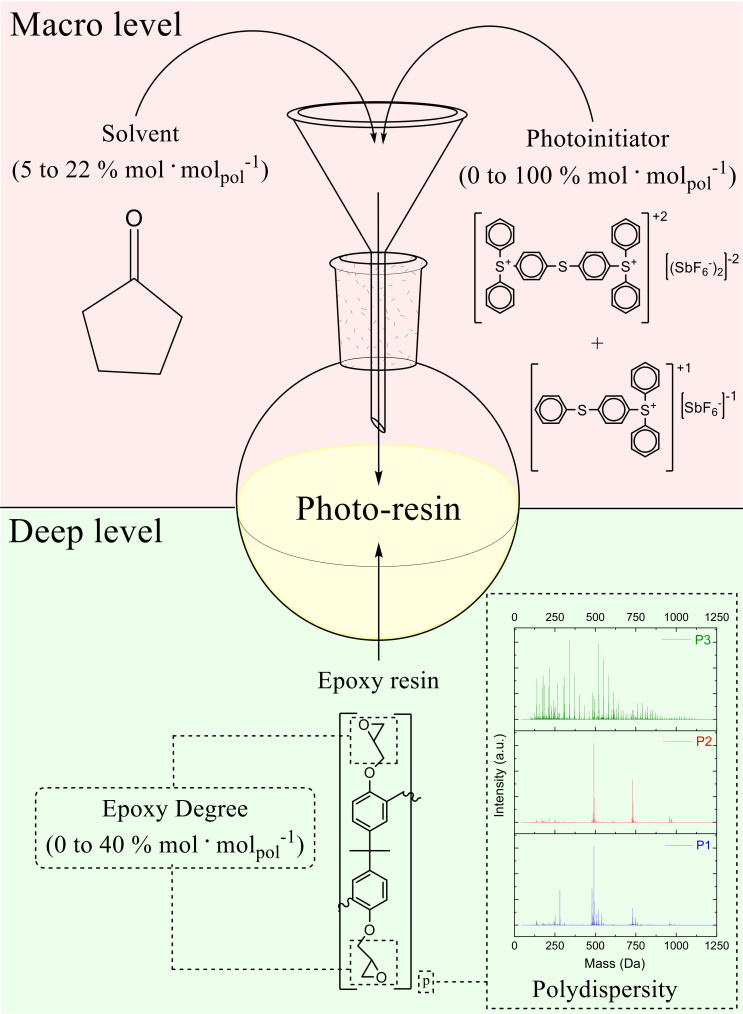
Deep and macro levels of properties for epoxy based photo-resins.

This work is focused on correlation between the chemical composition of the photo-resins and its lithographic performance applying deep-UV and UV lithography techniques. Macro level of properties was studied and assessed concerning only the three essential components: epoxy resins, photoinitiator, and solvent, as Figure 3 presents.

## 2. Materials and Methods

### 2.1. Materials

Chemicals were purchased from Sigma-Aldrich (Darmstadt, Germany). A mix of triarylsulfonium hexafluoroantimonate salts (TAS) (50 wt % in propylene carbonate) and solvents as isopropanol (IPA) (anhydrous, 99.5%), dimethyl sulfoxide (max. 0.025% H_2_O), cyclopentanone (99.8%) were used in the experiments. All the chemicals were used as received.

Aryl epoxy resins were synthesized and characterized previously. A detailed description of this step is presented by Vlnieska et al., (2019) [10]. Epoxy resins (poly(2,2-bis(4-oxy-(2-(methyloxirane)phenyl)propan) are oligomer chains distributed mainly in the range of dimers to tetramers, presenting in some cases traces of pentamers and hexamers in the distribution profile of the oligomeric chains. The averaged molar mass (M_n_) is 607 Da and polidispersity of 1.015. In this work they are named P1, P2, and P3 oligomer matrixes.

### 2.2. Methods

The characterization methods and lithographic procedures described are organized in chronological order of application.

#### 2.2.1. Quality Evaluation Using UV Lithography

**Figure 4:**
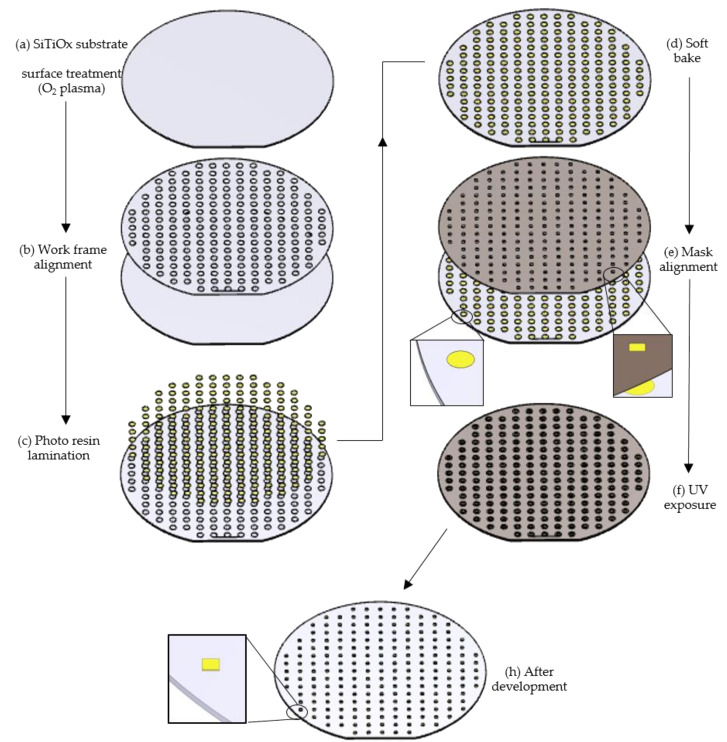
Qualitative evaluation of the photo-resins using 3 mm diameter and 120 µm thick cavities. In (**a**) a plasma treatment is applied in the choose substrate; (**b**) the substrate is aligned with the working frame; (**c**) depicts the lamination of the photoresins in the substrate with working frame; (**d**) represents the soft baking step to evaporate the solvent of the photoresin´s formulation; (**e**) shows the aligment of the exposure mask (in this case, UV irradiation); (**f**) depicts the substrate, laminated and aligned with the exposure mask, and (**h**) represents the final structures after development step and overnight drying.

Photo-resins with a variety of formulations (see Table 1) were evaluated in a qualitative manner to correlate the chemical composition of the photo-resins and its lithographic performance. The lithography process was performed in a cleanroom environment, with controlled temperature and humidity (23 °C, 45–50% H_2_O). Figure 4 presents the experimental methodology.

In Figure 4a, the used substrate was silicon titanium oxide (SiTiO_x_) (550 µm of thickness, the diameter of 4 inches (10.16 cm), and circa 2.5 µm of TiO_x_ layer in the surface). SiTiO_x_ substrates were pre-cleaned with plasma etching (PE) (see method in Section 2.2.3). After PE the substrate was laid on a heating plate at 95 °C for 10 min. Afterwards a work frame lamination was glued to the substrate (Figure 4b). The working frame is an adhesive itself. The features of the work frame are a circle shape with three millimeters diameter and approximately 120 µm height. The photo-resins were laminated in the work frame (Figure 4c). Using a tiny needle, small drops of photo-resins were placed in the circular shapes of the work-frame. After that, the photo-resins were leveled with the surface of the work frame by lamination with a small spatula. After lamination, the substrate was laid in a heating plate at 65 °C during 6 h (Figure 4d). In Figure 4e, a mask made of Kapton^®^ (120 µm thickness, with squared shapes of 2.3 mm^2^) was aligned with the work frame. After alignment, the photo-resins were exposed at flood exposure mode (Figure 4f), see Section 2.2.5. The post-exposure bake was made in the oven for 12 h at 65 °C (Figure 4g). The development was made using 2-methoxy-1-methyl ethyl acetate (PGMA) and isopropanol (ISO) as developers, following the order and time: 7 min in PGMA and 5 min in ISO. The wafers were laid at room temperature overnight to dry out the excess of the solvent.

#### 2.2.2. Test Pattern Structures Using Deep-UV and UV Lithography

Photo-resins (see Table 1) were evaluated in order to correlate the chemical composition of the photo-resins and its lithographic performance. The photo-resins were processed at the cleanroom facilities. Temperature and humidity were controlled (23 °C, 45–50% H_2_O). SiTiOx substrates (550 µm of thickness, the diameter of 1 cm and circa 2.5 µm of TiO_x_ layer in the surface) were pre-cleaned with PE (see Section 2.2.3). After PE the substrate was laid in a heating plate at 95 °C for 10 min. Substrates were coated with photo-resins using the spin-coating technique (see Section 2.2.4). After spin-coating, the substrates were laid in an oven at 65 °C for 6 h.

The coated substrates (1 cm diameter) were glued in a 4 inches (10.16 cm) diameter silicon substrate, using double-sided adhesive tape. The samples were placed in a holder from the EVG mask aligner equipment. The samples were exposed using deep-UV and UV light (see Section 2.2.5). After exposure, the samples were laid in an oven for 12 h at 65 °C. The development was made using 2-methoxy-1-methyl ethyl acetate (PGMA) and isopropanol (ISO) as developers, following the order and time of 7 min in PGMA and 5 min in ISO. The wafers were laid at room temperature overnight to dry out the excess of the solvent.

#### 2.2.3. Plasma Etching 

Silicon titanium oxide substrates were pre-cleaned with plasma etching. Plasma etching was processed at the cleanroom facilities. Temperature and humidity were controlled (23 °C, 45–50% H_2_O). Substrates were laid directly in the chamber of the equipment. The cleaning procedure was carried out in an equipment model, Etchlab 200, from Sentech (Berlin, Germany). The parameters for the plasma etching were: 100 W; 400 mTorr and 170 V in the reaction chamber; running time of 600 s.

#### 2.2.4. Spin-Coating

Spin-coating technique was applied to coat substrates with the photo-resin formulations. In the cleanroom environment, temperature and humidity were controlled (23 °C, 45–50% H_2_O). The samples were prepared as follows: at first, the substrates (SiTiOx, 550 µm, 1 cm diameter) were placed in the holder for the spin-coating equipment, then the vacuum-line was activated. A small amount of the samples were poured on the substrates. The spin-coating was performed in three steps to achieve a uniform 20–30 µm thickness: 1) spin-coating at the rotational speed of 1500 rpm with speed rate of 100 rpm·s^−1^ for 60 s, 2) 60 s rest, 3) spin-coating at 2750 rpm with the speed rate of 100 rpm·s^−1^ during 75 s.

#### 2.2.5. Deep-UV and UV Lithography Exposures

Substrates containing the photo-resins were exposed to deep-UV and UV light to evaluate the lithographic properties of the photo-resins and compare them with its formulations. The photo-resins were processed at the cleanroom environment. Temperature and humidity were controlled (23 °C, 45–50% H_2_O). The photo-resin layers were exposed using an EVG mask aligner (EV Group, Sankt Florian am Inn, Austria) with 2.8 mW·cm^−2^ radiation intensity (0.15 mW·cm^−2^ deviation) using the filter for wavelengths shorter than 365 nm. For the first set of experiments, a mask made of Kapton^®^ (125 µm of thickness) was used, with squares of 2.3 mm^2^. For the second set, a chromium mask was used as a test pattern, with dimensions of the structures varying from 50 down to 1 µm width. The samples and the mask were pre-mounted as a whole object, with contact between the mask and surface of the photo-resins. The dose delivered to the surface of the photoresist was 10 J·cm^−2^.

#### 2.2.6. Scanning Electron Microscopy (SEM)

The structures obtained after deep-UV and UV lithography were evaluated using scanning electron microscopy (SEM). The samples were glued with a special adhesive tape (for SEM measurement) on a silicon substrate (500 µm thick, with a 4 inches diameter (10.16 cm)), used as a base plate. The substrate was fixed in a holder for 4 inches substrate. The holder was inserted into the SEM chamber, and the measurements were obtained. The measurements were carried out using equipment model Zeiss Supra 60 VP from Carls Zeiss GmbH (Oberkochen, Germany). The energy of the beam used in the measurement was in the range from 0.7 to 10 keV.

#### 2.2.7. Optical Microscope Measurements

Laminated photo-resins with a variety of formulations (see Table 1) and spin coated samples (Tables S4 to S7 from Supplementary Materials) were evaluated using an optical microscope after the lithographic processing. The evaluation was made at the cleanroom facilities, with controlled temperature and humidity (23 °C, 45–50% H_2_O). Samples were placed in the table holder of the optical microscope. After focusing the cavities with the photo-resins formulations, the pictures were recorded. The measurements were conducted using a digital microscope VHX with the lenses model VH-Z 20R–RZ 20×–200×, from Keyence (Itasca, Minneapolis, MN, USA). The images were taken with magnifications varying from 175 to 200 times, with white light and no filters in the optical path.

## 3. Results and Discussion

### 3.1. Formulation of the Photo-Resins

The formulation or macro level preparation of a photo-resin involves several steps and chemical compounds to achieve the final formulation. Nevertheless, for chemically amplified formulations, the most straightforward possible composition must have at least three compounds: a polymer or oligomer, a photoinitiator, and a solvent. Additionally, to improve its mechanical properties and lithographic performance several additives are usually added to the formulations. Regarding epoxy resins, two characteristics were considered to be evaluated in the formulations: the polydispersity and the epoxidation degree. For the formulations cyclopentanone was used as a solvent, whose main function is to obtain a specific viscosity for the formulations (850 ± 20 mPa·s^−1^ (25 °C, 1 × 10^3^ s)).

The resins were studied applying the tuning of deep level properties. Three different profiles of polydispersity from the resins (P1, P2, and P3), and the epoxidation degrees, with concentration range from 0 to 40% mol·mol_polymer_^−1^ were evaluated in several formulations. A detailed study about the synthesis and characterization of the epoxy resins is presented by Vlnieska et al. [10]. Regarding tuning on the macro level of properties, the amount of photoinitiator was studied from 0 to 100% mol·mol_polymer_^−1^. Table 1 presents the studied properties for the photo-resins.

The initial experiments were prepared to combine the features presented in Table 1. To easily comprehend the formulations, the following code was used:

PxEDyCz

where: P: profile of the resin

ED: epoxidation degree
C: photoinitiator
x: type of resin
y: the amount of epoxy groups
z: the amount of catalyst

A quantity of 35 photo-resins (all “ED’s” multiplied by all “C´s” in Table 1) were formulated using initially only one type of epoxy resin. Using UV lithography, a qualitative experiment (see Section 2.2.4) was performed to evaluate the performance of the epoxidation degree and the amount of catalyst.

### 3.2. Quality Evaluation of the Photo-Resins

**Figure 5:**
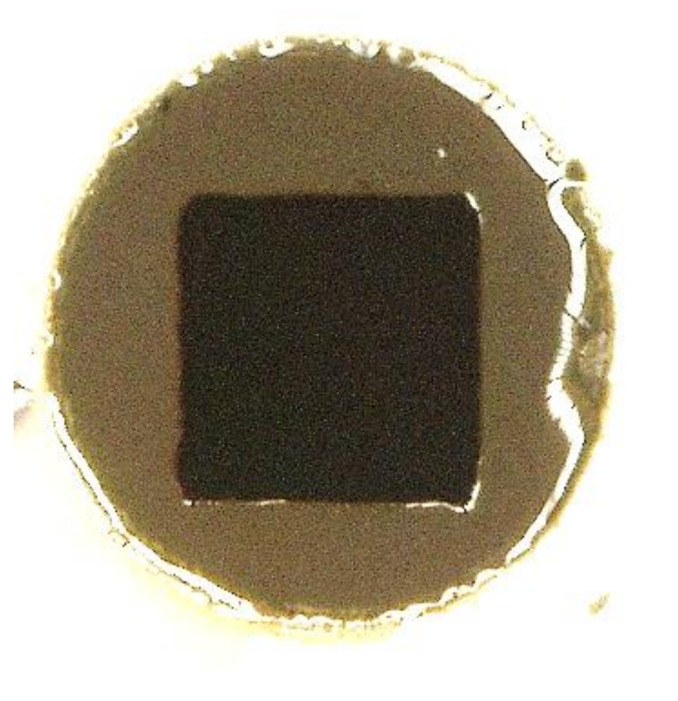
Optical microscope image from the formulation P_2_ED_0_C_3_ (t3) (Table S1).

In the Supplementary Materials, it is possible to observe Figures SF1 to SF3, where the substrates (SiTiOx–500 µm thickness, four inches diameter) are prepared with the framework, laminated, and soft baked with the formulations. Table S1 shows the photo-resins after UV exposure and post-exposure bake. In Table S1, it is interesting to note the pattern of the structures straightforward after the post exposure bake (PEB). Starting with 3% mol∙mol_polymer_^−1^, all samples presented a darker tone color in the exposed region (when it is compared with the unexposed region). Figure 5 exemplifies this characteristic.

Although in Figure 5 it is not possible to see the depth of the structure, the difference in the tones between exposed and non-exposed areas allow the observation of the pattern contours. It can help to decide if the lithography process should proceed to the next step or not. As can be observed in Table S1, the formulations without epoxy groups (P_2_ED_x_C_0_) seem to result in poor adhesion to the substrates. Formulations with high concentrations of photoinitiator (C_50_ and C_100_) combined with low epoxidation degree (ED_0_ and ED_10_) also lead to poor results for most of these formulations (imperfections in the structures or low adhesion to the substrate).

Table 2 presents the appearance of the photo-resins after the development step. The quality criteria were assigned observing the structures at an optical microscope (see Section 2.2.7). Due to the large number of samples (each formulation was evaluated in triplicates, resulting in a set of 105 samples) the first coarse selection was performed considering the overall criteria:

- 0 value for the not remained structure after the development step (complete lift-off);
- 0.5 value for remained structures with defects after the development;
- 1.0 value for entirely remained structures after the development.

**Figure 6:**
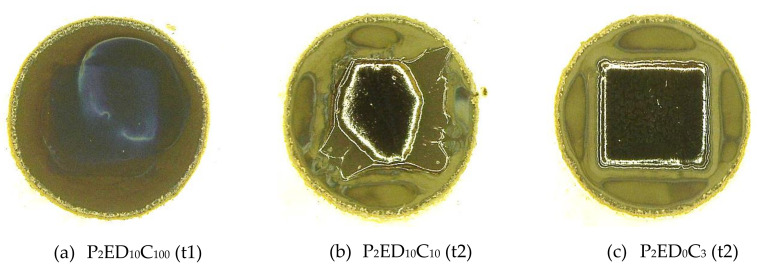
Criteria used in the quality evaluation of the photo-resins corresponding to: (**a**) 0, (**b**) 0.5, and (**c**) 1.0 values.

Figure 6 illustrates the quality criteria which were assigned with the above-mentioned values.

In Figure 6a, the value 0 was given to the lithographic performance of P_2_ED_10_C_100_ (t1), where the formulation was completely removed after development. Figure 6b presents an example of a partially remained structure, which was assigned the value of 0.5. In Figure 6c, the structure entirely remained, receiving the value 1. In the Supplementary Materials, the microscope images from all samples after development step are presented in Table S2. In Table S3 the set of values given to the images to assess the quality of the squared patterns is presented.

**Figure 7:**
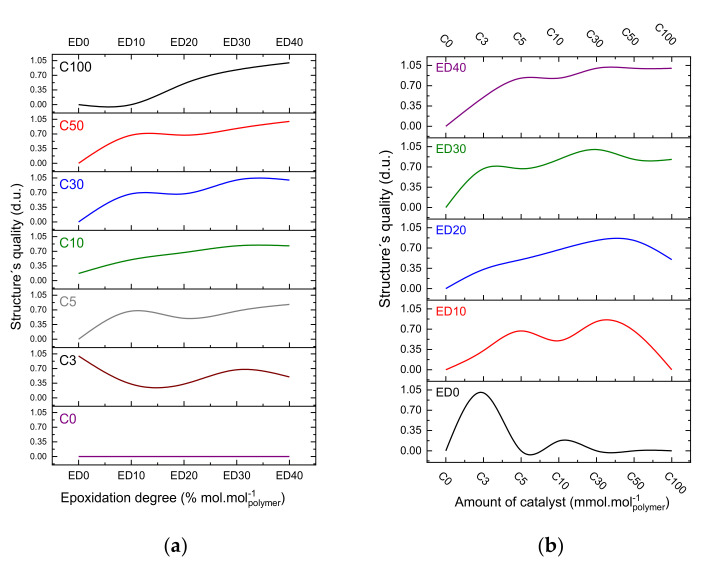
Epoxidation degree × amount of photoinitiator: (**a**) emphasizing the photoinitiator; (**b**) emphasizing the epoxidation degree.

**Figure 8:**
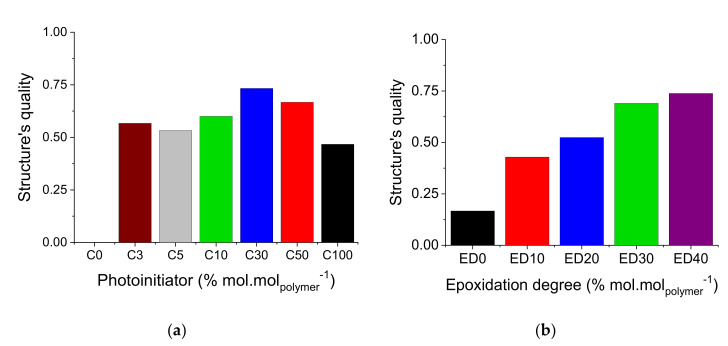
Averaged performance related to (**a**) the amount of photoinitiator; (**b**) the epoxidation degree.

Figure 7 and Figure 8 present the results calculated in Table S3, which shows the influence of the epoxidation degree and the photoinitiator concentrations on the quality of the obtained structures. Figure 7a and Figure 8a present the results emphasizing the amount of photoinitiator in the formulations, whereas the Figure 7b and Figure 8b emphasize the epoxidation degree in the formulations. In general, the formulations start to give reliable results with concentrations beginning with 30% mol·mol_polymer_^−1^ for epoxidation degree and amount of catalyst. One can see an interesting result regarding the curve in C_3_ (Figure 7a), where it was possible to achieve structures with good quality with no epoxy content in the formulation.

In Figure 7b one can see that the formulation with epoxidation degree of 40 % mol·mol_polymer_^−1^ appears the most stable, producing structures of good quality with amounts of photoinitiator from 5 to 100% mol·mol_polymer_^−1^ (range of values from 0.7 to 1.0). Figure 8a,b represent a comparison between formulations using the averaged results for each concentration. For example, in Figure 8b the point that represents ED_30_ is the average of all triplicates and concentrations tested with epoxidation degree of 30% mol·mol_polymer_^−1^. Figure 8a illustrates that the best results were achieved with 30 and 50% mol·mol_polymer_^−1^, regarding the amount of catalyst. In Figure 8b, one can see that the best performance is achieved with concentrations of 30 and 40% mol·mol_polymer_^−1^, regarding the epoxidation degree. The epoxidation degree of 40% mol·mol_polymer_^−1^ has slightly better performance.

**Figure 9:**
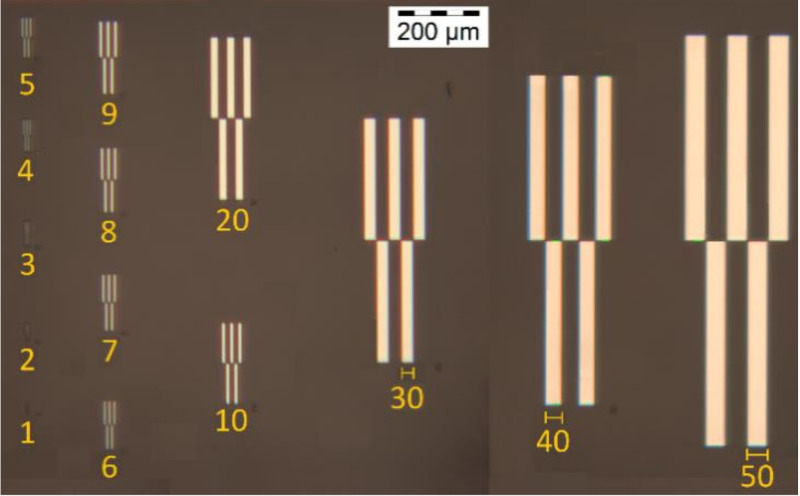
Design and pitch of the UV mask.

The results from the quality evaluation indicate the P_x_ED_40_C_30_ as the appropriate formulation to proceed with further investigation. This formulation was prepared using three profiles of resins, P1, P2 and P3, resulting in P_1_ED_40_C_30_, P_2_ED_40_C_30_, and P_3_ED_40_C_30,_ which were processed using UV and deep-UV lithography techniques (see Section 2.2.5), using a pattern test mask with feature sizes from 50 to 1 µm width. Figure 9 presents the design of the chromium mask (the picture was made with an optical microscope; see method in Section 2.2.6).

Tables S2–S5 present the microscope images from the mentioned formulations and their triplicates. The best results were evaluated by SEM. Table 2 compares the chemical composition of the formulations with the final structures. SEM images from Table 2 are available in full format in the Supplementary Materials (Figures SF4–SF6).

As shown in Table 2, in general, good quality structures were obtained from 50 down to 10 µm width. Since the thickness of the photo-resins was in the range of 30 µm, the fabrication of sharp structures with lateral dimensions smaller than 10 µm width is challenging even for commercial formulations of photo-resins, observed previously in the work of Zakharova et al. (2018), where rectangular patterns were studied [14]. Comparing the formulations one can see a better sharpness of the structures when the formulation P_1_ED_40_C_30_ is utilized. It is interesting to note that for deep-UV lithography the formulations P_x_ED_40_C_30_ presented overexposed structures, which can be suggested by the fringes and disruptions in the structures. This result was expected since the exposure dose is considered too high for UV and deep-UVL [15,16,17]. The intention of this evaluation was initially to use the same irradiation doses for UV and deep-UV wavelengths, which was in both cases 10 J∙cm^2^. Regarding UV lithography, the best result was achieved with the P1 resin, where the polydispersity is the intermediate one. In this case, one can see a better photosensitivity and resolution of the structures. It is possible to observe that the limit of the resolution for the proposed formulations reaches 10 µm structure width. Structures smaller than this width size were not achieved, which can be related to diffraction effects or low photosensitivity of the formulations.

One can see in Table 2 the SEM pictures of the structures fabricated with UV lithography. The sharpest structures were obtained using the formulation P_1_ED_40_C_30_. The SEM evaluation for all samples was made in the same measurement with exactly the same parameters (method in Section 2.2.6).

**Figure 10:**
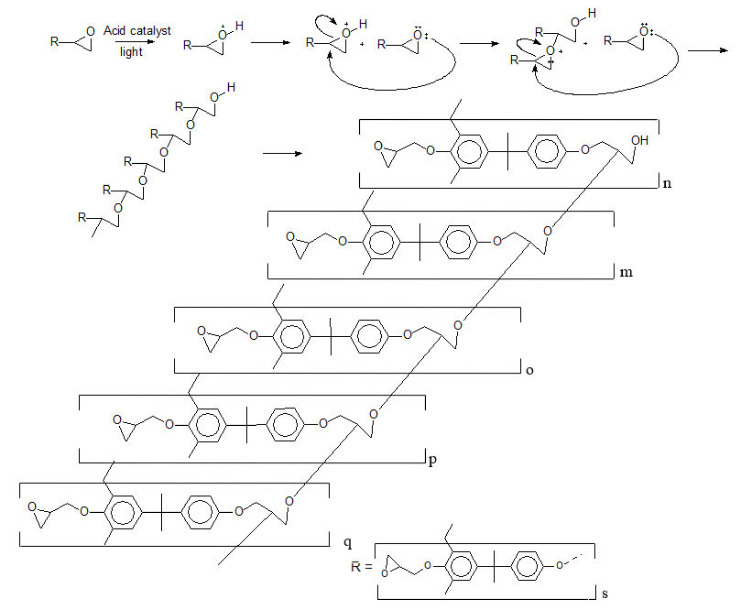
Crosslinking reaction mechanism for aryl-epoxy photo-resins.

For this type of epoxy resin, the cross-linking reaction that occurs during the exposure step is open to discussion in the literature and not completely elucidated. Figure 10 describes the crosslinking mechanism, considering the simplest reaction path. Consequently, a few constraints were assumed to understand the reaction mechanism, which are:

(1) The photoinitiator is a Lewis acid, releasing a proton to the reaction medium;
(2) The oligomers (Poly(2,2-Bis(4-hydroxyphenyl)propane)) have no secondary products and termination groups [10];
(3) All the epoxy rings are intact, and there is no epoxy derivate [10]; and
(4) The epoxidation degree of poly(2,2-Bis(4-hydroxyphenyl)propane) is 100%.

Once the photoinitiator is activated with light (UV light in this case), a proton is released in the medium and initiates a cascade reaction in the epoxy groups. Assuming that the epoxy groups are close enough to one each other, the cascade reaction go through the polymer chains (“n”, “m”, “o”, “p”, “q”), bounding the oligomers, which leads to a rigid and permanent solid state structure. Comparing the polydispersity and SEM pictures (rows 1 and 4–Table 2), it is reasonable to suggest:

(1) The polydispersity of the photo-resins shall be considered in crosslinking mechanism; and
(2) The crosslinking efficiency (irradiation step) tend to be improved if the oligomer chains present less secondary and derivative products.

**Figure 11:**
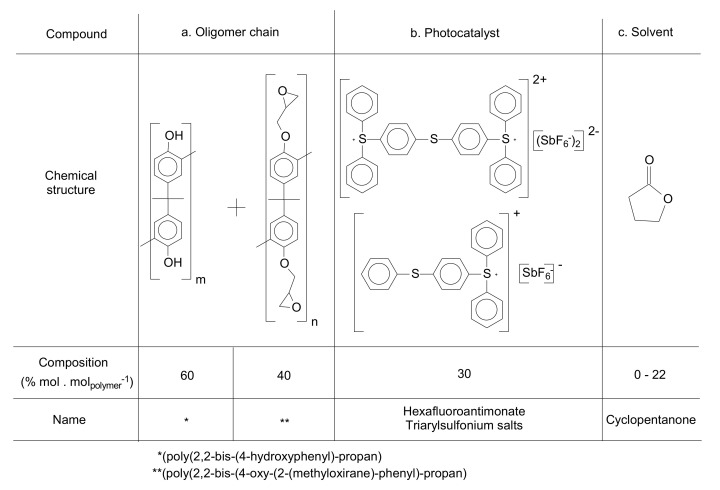
Best epoxidation degree composition for UV lithography.

Regarding the epoxidation degree, Figure 11 presents the composition of the photo-resins studied in the second set of experiments. For the oligomer composition, it is crucial to mention that this representation is simplified and does not take into consideration the ending and secondary groups from the oligomer´s chains as well as the secondary products from the epoxidation degree [10].

## 4. Conclusions

Photo-resins were formulated based on the deep and macro levels of properties. For this purpose, previously synthesized and characterized resins were applied. Two series of experiments were conducted, both in a qualitative manner. The photo-resins were prepared using the most basic formulation possible regarding the macro level of properties, employing the epoxy resins, a photoinitiator (TAS), and cyclopentanone as a solvent.

In the first set of experiments, UV lithography showed that good quality structures (values from 0.7 to 1.0 shown in Figure 7a,b could be achieved using a broad range of concentrations for the epoxidation degree and photoinitiator. In this case, the photo-resins can be formulated with an epoxidation degree from 10% mol·mol_polymer_^−1^ to 40% mol·mol_polymer_^−1^. Regarding the photoinitiator, the concentration can vary from 3% mol·mol_polymer_^−1^ to 100% mol·mol_polymer_^−1^. It seems that the tuning of deep and macro levels of properties is not significant to improve the quality of large structures. A few assumptions can be suggested to explain the results: for a large volume of the structure, the adhesion between the photo-resin and the substrate is less critical compared with sub-micrometer structures; the oligomer chains most probably do not need to be totally crosslinked; consequently, less amount of photoinitiator is necessary for the formulation.

In the second set of experiments regarding UV lithography, structures from 50 to 10 µm width were produced using the photo-resins. In this case, a few characteristics of the formulations were observed. Concentrations of epoxy degree and photoinitiator presented better results in a narrow range of composition, with respectively 40% mol·mol_polymer_^−1^ and 30% mol·mol_polymer_^−1^. The polydispersity of the epoxy resins starts to play a significant role for structures smaller than 50 µm. In this case, formulations with epoxy resin P1 presented better quality in the structures compared with the other two resins (P2 and P3). For DUV lithography, a behavior similar to the overexposure effects was observed. This effect was expected since the initial approach was to compare the same irradiation dose for both techniques. The best result was achieved with P_1_ED_40_C_30_ formulation.

Observing the results, two main statements can be proposed:

(1) For large structures (roughly above 1 mm^2^), the tuning of the deep and macro levels of properties can be less restricted;
(2) For structures under 50 µm it is necessary to consider a specific polydispersion profile of the photo-resin to achieve better quality in the structures (deep level of properties). It is also suggested to restrict the concentrations for the epoxidation degree (deep level of properties), and photoinitiator (macro level of properties).

This study aimed to achieve a proof of concept approach. It is confirmed that the chemical composition within the deep level of properties is directly related to the quality of the obtained structures, mainly regarding feature sizes below 50 µm. It was possible to observe significant changes in the quality of the structures using the most basic formulation, without having extra additives. However, the overall patterning quality depends not only on the suitable photoresist formulation but also a significant contribution is given by the process parameters. Currently, the optimal spin coating, baking, exposure, and development parameters are under investigation for the proposed formulations using UVL and DUVL.

## Figures and Tables

**Table 1:** Chemical compounds, oligomer characteristics and its quantities for the photo-resin formulations.

Level of Formulation	Compound	Range (% mol·mol_polymer_^−1^)
Deep level (oligomer composition)	Profile of Resin (polydispersity)	P1, P2, P3
	Epoxidation degree	0; 10; 20; 30; 40
Macro level (added chemicals to the formulation)	Photoinitiator	0; 3; 5; 10; 30; 50; 100
	Solvent	0–22 *

* the solvent concentration varies to reach the right viscosity.

**Table 2:** Comparison between formulations, using mass spectroscopy, optical, and SEM microscopy of the patterned structures.

	P_1_ED_40_C_30_	P_2_ED_40_C_30_	P_3_ED_40_C_30_
Polydispersity (Mass spectroscopy)	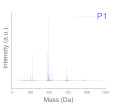	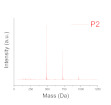	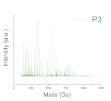
Optical microscopy (UVL)	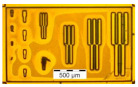	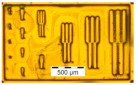	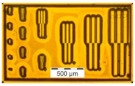
Optical microscopy (DUVL)	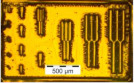	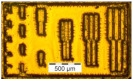	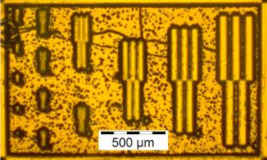
Scanning electron microscopy (UVL)	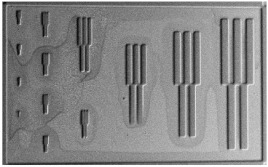	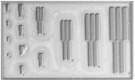	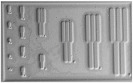

## Supplementary Materials

The following are available online at https://www.mdpi.com/2073-4360/12/10/2359/s1; Figure SF1. Triplicate 1 of the quality evaluation; Figure SF2. Triplicate 2 of the quality evaluation; Figure SF3. Triplicate 3 of the quality evaluation; Figure SF4. SEM images of P_1_ED_40_C_30_ formulation. Patterned structures generated thorough UV lithography after development; Figure SF5. SEM images of P_2_ED_40_C_30_ formulation. Patterned structures generated thorough UV lithography after development; Figure SF6. SEM images of P3ED40C30 formulation. Patterned structures generated thorough UV lithography after development. Table S1. Images of the photo-resin formulations after lamination, UV-exposure, and hard bake; Table S2. Images of the photo-resin formulations after development; Table S3. Quality control for the evaluation of the photo-resin formulations; Table S4. Patterned structures generated thorough UV lithography after hard bake; Table S5. Patterned structures generated thorough deep-UV lithography after hard bake; Table S6. Patterned structures generated thorough UV lithography after development; Table S7. Patterned structures generated thorough deep-UV lithography after development.
