# Supplementary material for: Lithographic Performance of Aryl Epoxy Thermoset Resins as Negative Tone Photoresist for Microlithography
Source: Polymers (Basel). 2020 Oct 14;12(10):2359. doi: 10.3390/polym12102359 (PMC7650645; doi:10.3390/polym12102359)
Supplement: Supplementary file 1 [file polymers-12-02359-s001.zip › polymers-931848-supplementary.docx]

***Supplementary Material***

| 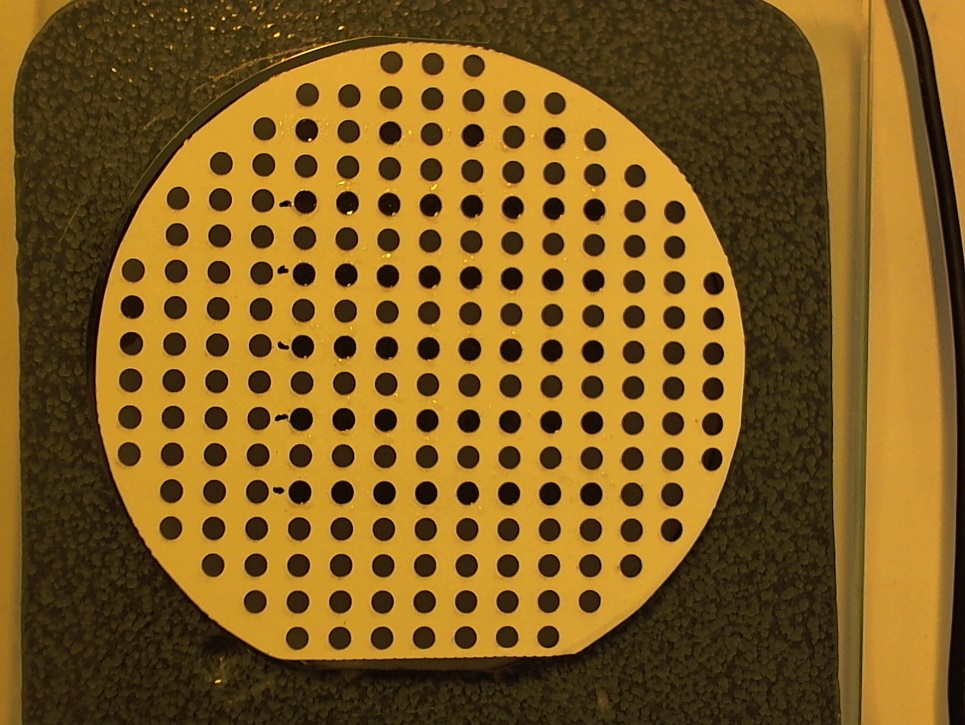  **Figure SF1:** Triplicate 1 of the quality evaluation. | 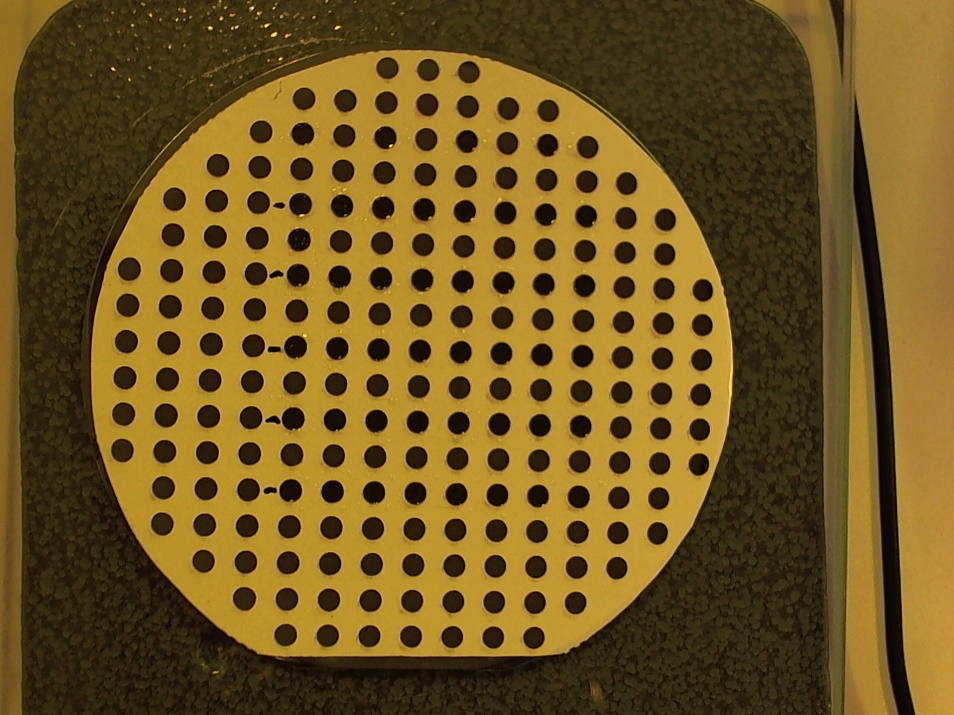  **Figure SF2:** Triplicate 2 of the quality evaluation. | 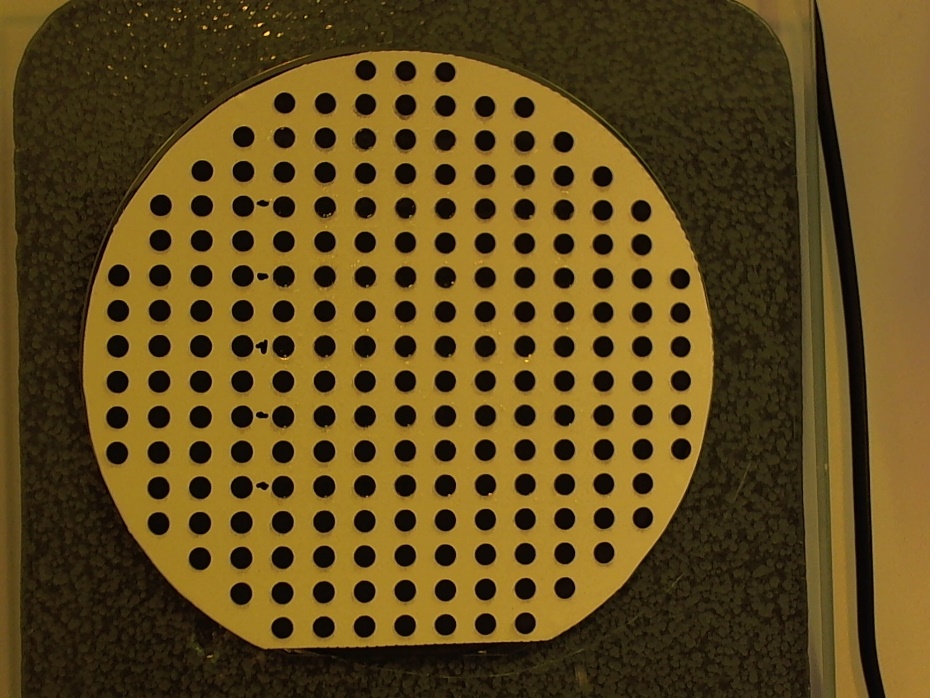  **Figure SF3:** Triplicate 3 of the quality evaluation. |
| --- | --- | --- |

**Table S1:** Images of the photo-resin formulations after lamination, UV-exposure, and hard bake.

|  | ED_0_ | | | ED_10_ | | | ED_20_ | | | ED_30_ | | | ED_40_ | | |
| --- | --- | --- | --- | --- | --- | --- | --- | --- | --- | --- | --- | --- | --- | --- | --- |
|  | T_1_ | T_2_ | T_3_ | T_1_ | T_2_ | T_3_ | T_1_ | T_2_ | T_3_ | T_1_ | T_2_ | T_3_ | T_1_ | T_2_ | T_3_ |
| C_0_ | 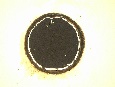 | 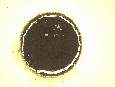 | 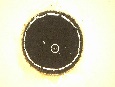 | 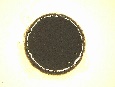 | 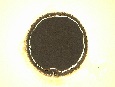 | 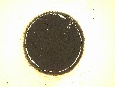 | 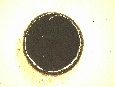 | 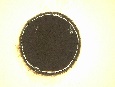 | 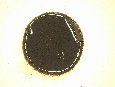 | 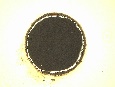 | 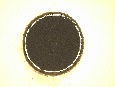 | 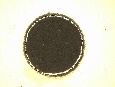 | 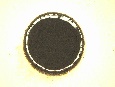 | 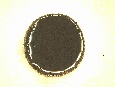 | 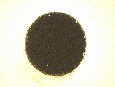 |
| C_3_ | 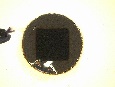 | 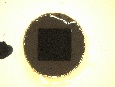 | 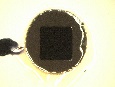 | 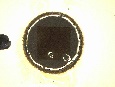 | 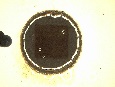 | 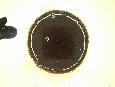 | 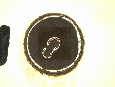 | 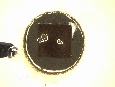 | 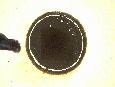 | 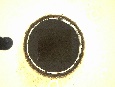 | 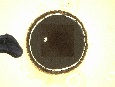 | 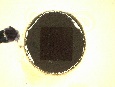 | 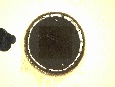 | 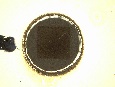 | 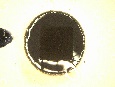 |
| C_5_ | 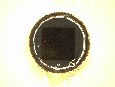 | 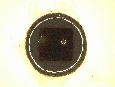 | 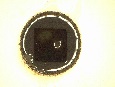 | 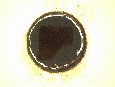 | 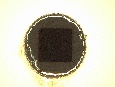 | 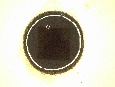 | 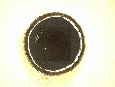 | 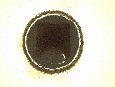 | 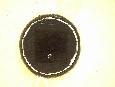 | 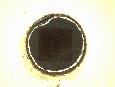 | 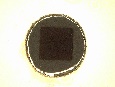 | 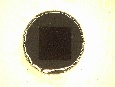 | 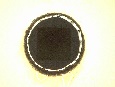 | 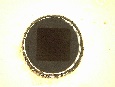 | 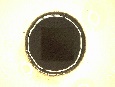 |
| C_10_ | 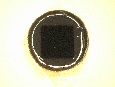 | 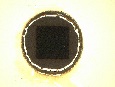 | 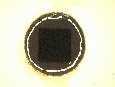 | 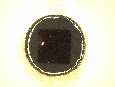 | 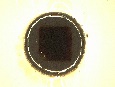 | 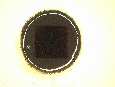 | 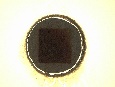 | 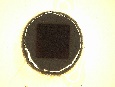 | 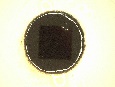 | 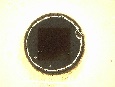 | 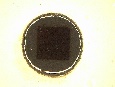 | 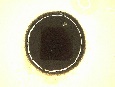 | 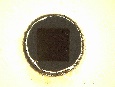 | 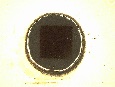 | 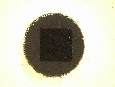 |
| C_30_ | 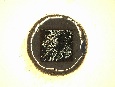 | 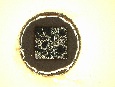 | 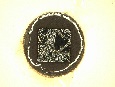 | 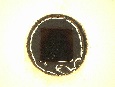 | 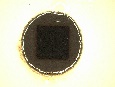 | 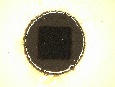 | 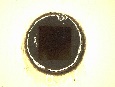 | 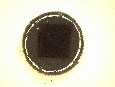 | 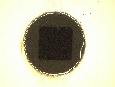 | 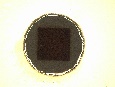 | 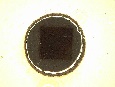 | 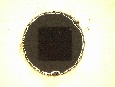 | 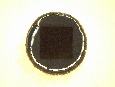 | 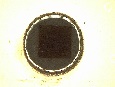 | 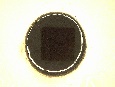 |
| C_50_ | 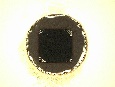 | 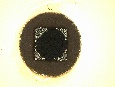 | 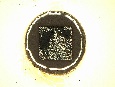 | 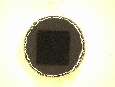 | 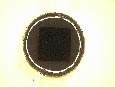 | 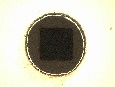 | 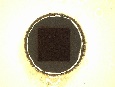 | 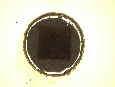 | 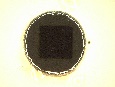 | 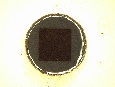 | 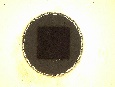 | 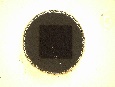 | 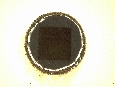 | 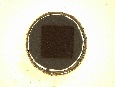 | 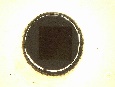 |
| C_100_ | 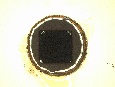 | 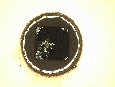 | 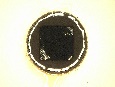 | 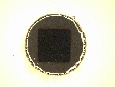 | 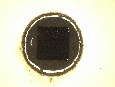 | 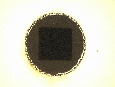 | 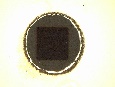 |  |  |  |  |  |  |  |  |

ED = Epoxidation degree (% mol∙(mol_polymer_)^-1^)

C = Amount of photocatalyst (% mol∙(mol_polymer_)^-1^)

T = triplicate experiment 1, 2 and 3

**Table S2:** Images of the photo-resin formulations after development.

|  | ED_0_ | | | ED_10_ | | | ED_20_ | | | ED_30_ | | | ED_40_ | | |
| --- | --- | --- | --- | --- | --- | --- | --- | --- | --- | --- | --- | --- | --- | --- | --- |
|  | T_1_ | T_2_ | T_3_ | T_1_ | T_2_ | T_3_ | T_1_ | T_2_ | T_3_ | T_1_ | T_2_ | T_3_ | T_1_ | T_2_ | T_3_ |
| C_0_ |  |  |  |  |  |  |  |  |  |  |  |  |  |  |  |
| C_3_ |  |  |  |  |  |  |  |  |  |  |  |  |  |  |  |
| C_5_ |  |  |  |  |  |  |  |  |  |  |  |  |  |  |  |
| C_10_ |  |  |  |  |  |  |  |  |  |  |  |  |  |  |  |
| C_30_ |  |  |  |  |  |  |  |  |  |  |  |  |  |  |  |
| C_50_ |  |  |  |  |  |  |  |  |  |  |  |  |  |  |  |
| C_100_ |  |  |  |  |  |  |  |  |  |  |  |  |  |  |  |

ED = Epoxidation degree (% mol∙(mol_polymer_)^-1^)

C = Amount of photocatalyst (% mol∙(mol_polymer_)^-1^)

T = triplicate experiment 1, 2 and 3

**Table S3:** Quality control for the evaluation of the photo-resin formulations.

| **Samples** | **ED0 (t1)** | **ED0 (t2)** | **ED0(t3)** | **ED10(t1)** | **ED10(t2)** | **ED10(t3)** | **ED20(t1)** | **ED20(t2)** | **ED20(t3)** | **ED30(t1)** | **ED30(t2)** | **ED30(t3)** | **ED40(t1)** | **ED40(t2)** | **ED40(t3)** | **C average** |
| --- | --- | --- | --- | --- | --- | --- | --- | --- | --- | --- | --- | --- | --- | --- | --- | --- |
| C0 | 0 | 0 | 0 | 0 | 0 | 0 | 0 | 0 | 0 | 0 | 0 | 0 | 0 | 0 | 0 | - |
| **C0 t-average** | **0.0** | | | **0.0** | | | **0.0** | | | **0.0** | | | **0.0** | | | **0.0** |
| C3 | 1.0 | 1.0 | 1.0 | 0.0 | 0.5 | 0.5 | 0.0 | 0.5 | 0.5 | 0.5 | 0.5 | 1.0 | 0.5 | 0.5 | 0.5 | - |
| **C3 t-average** | **1.0** | | | **0.3** | | | **0.3** | | | **0.7** | | | **0.5** | | | **0.6** |
| C5 | 0.0 | 0.0 | 0.0 | 0.5 | 1.0 | 0.5 | 0.5 | 0.5 | 0.5 | 0.5 | 1.0 | 0.5 | 0.5 | 1.0 | 1.0 | - |
| **C5 t-average** | **0.0** | | | **0.7** | | | **0.5** | | | **0.7** | | | **0.8** | | | **0.5** |
| C10 | 0.5 | 0.0 | 0.0 | 0.5 | 0.5 | 0.5 | 0.5 | 1.0 | 0.5 | 0.5 | 1.0 | 1.0 | 0.5 | 1.0 | 1.0 | - |
| **C10 t-average** | **0.2** | | | **0.5** | | | **0.7** | | | **0.8** | | | **0.8** | | | **0.6** |
| C30 | 0.0 | 0.0 | 0.0 | 0.5 | 1.0 | 1.0 | 1.0 | 0.5 | 1.0 | 1.0 | 1.0 | 1.0 | 1.0 | 1.0 | 1.0 | - |
| **C30 t-average** | **0.0** | | | **0.8** | | | **0.8** | | | **1.0** | | | **1.0** | | | **0.7** |
| C50 | 0.0 | 0.0 | 0.0 | 1.0 | 0.5 | 0.5 | 1.0 | 0.5 | 1.0 | 0.5 | 1.0 | 1.0 | 1.0 | 1.0 | 1.0 | - |
| **C50 t-average** | **0.0** | | | **0.7** | | | **0.8** | | | **0.8** | | | **1.0** | | | **0.7** |
| C100 | 0.0 | 0.0 | 0.0 | 0.0 | 0.0 | 0.0 | 0.5 | 0.5 | 0.5 | 0.5 | 1.0 | 1.0 | 1.0 | 1.0 | 1.0 | - |
| **C100 t-average** | **0.0** | | | **0.0** | | | **0.5** | | | **0.8** | | | **1.0** | | | **0.5** |
| **ED average** |  | **ED0** | **0.2** |  | **ED10** | **0.4** |  | **ED20** | **0.5** |  | **ED30** | **0.7** |  | **ED40** | **0.7** | - |

*t-average: the average values for the triplicate measurements

* Average: the average of the referred feature

**Table S4:** Patterned structures generated thorough UV Lithography after hard bake.

| Triplicate | P_1_ED_0_C_3_ | P_1_ED_40_C_30_ | P_2_ED_0_C_3_ | P_2_ED_40_C_30_ | P_3_ED_0_C_3_ | P_3_ED_40_C_30_ |
| --- | --- | --- | --- | --- | --- | --- |
| T1 |  |  |  |  |  |  |
| T2 |  |  |  |  |  |  |
| T3 |  |  |  |  |  |  |

**Table S5:** Patterned structures generated thorough Deep-UV Lithography after hard bake.

| Triplicate | P_1_ED_0_C_3_ | P_1_ED_40_C_30_ | P_2_ED_0_C_3_ | P_2_ED_40_C_30_ | P_3_ED_0_C_3_ | P_3_ED_40_C_30_ |
| --- | --- | --- | --- | --- | --- | --- |
| T1 |  |  |  |  |  |  |
| T2 |  |  |  |  |  |  |
| T3 |  |  |  |  |  |  |

**Table S6:** Patterned structures generated thorough UV Lithography after development.

| Triplicate | P_1_ED_0_C_3_ | P_1_ED_40_C_30_ | P_2_ED_0_C_3_ | P_2_ED_40_C_30_ | P_3_ED_0_C_3_ | P_3_ED_40_C_30_ |
| --- | --- | --- | --- | --- | --- | --- |
| T1 |  |  |  |  |  |  |
| T2 |  |  |  |  |  |  |
| T3 |  |  |  |  |  |  |

**Table S7:** Patterned structures generated thorough Deep-UV Lithography after development.

| Triplicate | P_1_ED_0_C_3_ | P_1_ED_40_C_30_ | P_2_ED_0_C_3_ | P_2_ED_40_C_30_ | P_3_ED_0_C_3_ | P_3_ED_40_C_30_ |
| --- | --- | --- | --- | --- | --- | --- |
| T1 |  |  |  |  |  |  |
| T2 |  |  |  |  |  |  |
| T3 |  |  |  |  |  |  |

**Figure SF4:** SEM Images of P_1_ED_40_C_30_ formulation. Patterned structures generated thorough UV Lithography after development.

**Figure SF5:** SEM Images of P_2_ED_40_C_30_ formulation. Patterned structures generated thorough UV Lithography after development.

**Figure SF6:** SEM Images of P_3_ED_40_C_30_ formulation. Patterned structures generated thorough UV Lithography after development.
